# P054: Prevalence and risk factor analysis for methicillin-resistant Staphylococcus aureus colonization in an acute care hospital

**DOI:** 10.1186/2047-2994-2-S1-P54

**Published:** 2013-06-20

**Authors:** ML Oh, SY Tan

**Affiliations:** 1 Infectious Diseases, Changi General Hospital, Singapore, Singapore

## Introduction

Methicillin-resistant Staphylococcus aureus (MRSA) infections have been associated with increased mortality and hospital costs. Active surveillance cultures (ASCs) for MRSA and aggressive contact precautions have been shown to reduce MRSA transmission. Universal screening incurs financial and physical resources.

## Objectives

To determine the prevalence of MRSA colonization at admission and to identify risk factors associated with MRSA colonization in adult patients.

## Methods

This study was conducted in 2 wards (one medical and one surgical, each 44 bedded) in Changi General Hospital. ASCs were performed from 20 Jan 2010 to 7 Jul 2010 on all patients admitted to these wards. ASC specimens consisted of one swab from the nares and another from axilla/groin. A random sample of MRSA-positive and MRSA-negative patients were reviewed for demographics and risk factors for MRSA colonization.

## Results

A total of 2090 patients were screened on admission. 129 medical and 93 surgical patients were MRSA positive on entry (total 222, 10.6%).

136 MRSA-positive patients were randomly selected and analyzed for risk factors for MRSA colonization. The mean age was 67.3 years (77.1% ≥ 60 years old) and average length of stay was 19 days. Among the 136, patients, 14% had urinary catheter, 38.2% diabetes, 17.6% malignancy, 5.1% chronic kidney disease and 14/7% had skin ulcer. 48 MRSA-negative patients were randomly selected and analysed as a control group. The mean age was 57.9 years and average length of stay was 13.6 days. Among the 48 patients, 4.2% had urinary catheter, 20.8% diabetes, 14.6% malignancy, 4.2% chronic kidney disease and 6.3% had skin ulcer.

Significant risk factors for MRSA colonization at admission included residence in a long term care facility, previous MRSA infection or colonization and diabetes mellitus. The majority of MRSA-positive patients were >60 years and had prolonged hospitalization.

## Conclusion

The prevalence of MRSA colonization was 10.6%. Risk factors for MRSA colonization included residence in a long term care facility, diabetes and previous MRSA colonization/infection. This study revealed the high burden of MRSA in Singapore. Knowledge of risk factors for MRSA colonization offer selective screening for MRSA based on risk factors as a more cost-effective strategy in reducing MRSA transmission.

## Disclosure of interest

None declared

